# The role of femoral head size in metal-on-metal hip arthroplasty: analysis of a cohort of 3813 patients with long term follow-up

**DOI:** 10.1007/s00402-024-05567-0

**Published:** 2024-11-05

**Authors:** Corrado Ciatti, Luca Andriollo, Chiara Asti, Davide Morsia, Fabrizio Quattrini, Monica Cosentino, Barbara Bordini

**Affiliations:** 1 University of Parma, Parma, Italy; 2 Orthopaedics and Traumatology Department, Guglielmo da Saliceto Hospital, Via Taverna 49, Piacenza, Italy; 3 Sezione di Chirurgia Protesica ad Indirizzo Robotico - Unità di Traumatologia dello Sport, Fondazione Poliambulanza Istituto Ospedaliero, Brescia, Italy; 4 Medical Technology Laboratory, IRCCS Istituto Ortopedico Rizzoli, Bologna, Italy; 5 Università Cattolica del Sacro Cuore, Roma, Italy; 6 Artificial Intelligence Center, Alma Mater Europea University, Vienna, Austria

**Keywords:** Total hip arthroplasty, Metal-on-metal, Head size, Long term follow-up, Arthroplasty registry

## Abstract

Metal-on-metal (MoM) large headed arthroplasties were suggested to decrease failure rates by means of reduced volumetric wear and enhanced implant stability. However, they caused high rates of revisions due to adverse reaction to metal debris, osteolysis, pseudotumor growth, and other issues. The study aims to present the long-term outcomes of MoM arthroplasties on a large cohort of patients, evaluating the impact of head sizes on survival rate. All data were retrieved from the regional joint register (Registro dell’Implantologia Protesica Ortopedica, RIPO, Italy). We include in the study all patient who underwent cementless MoM total hip arthroplasties (THAs) between 2000 and 2020, dividing them in two subgroups according to head size (<36 mm, ≥36 mm). Failures were recorded up to December 31, 2020. Patients lost to follow-up were excluded. A total of 3813 THAs met the inclusion/exclusion criteria. The average follow-up period is 12.4 years (0–21 years). 178/1625 (or 11.0%) small head MoM THAs and 265/2188 large head ones failed by the end of follow up. Large diameter heads reported lower survival rate (*p*-value < 0.001), with unexpected higher dislocation rate (1.0 vs. 0.4%). Moreover, large head size was found to increases the risk of metallosis (*p*-value < 0.0001). Gender, patient’s age and the use of modular neck were not correlated with higher failure rates. MoM implants implants do not have the same reliability as other couplings, considering the significantly greater failure/complication rates. However, the knowledge of risks linked to head size is fundamental for establishing the right type of follow-up to the patient and recognize any complications early.

## Introduction

Total hip arthroplasty (THA) is considered a successful orthopedic procedure for the treatment of hip pathologies [1]. The most common indication for THA is end-stage hip osteoarthritis (OA), but other widespread underlying pathologies are hip osteonecrosis and congenital or inflammatory diseases.

As a consequence of the increasing prevalence of THA, the amount of revision procedures is growing. The cause of the primary hip implant failure influences the outcome of the revision procedure and can lead to systemic pathological complications [2].

In the late 1990s and early 2000s, complications arising from polyethylene wear-induced osteolysis, component loosening, and wear in young patients with THAs led to the revival of components with cobalt-chromium metal-on-metal (MoM) bearing surfaces. These implants had been previously used until the mid-1970s, after which they were discontinued due to issues with prosthetic loosening, metallic debris, metal hypersensitivity, and theoretical risks of cancer [3].

This new generation of MoM THA implants was suggested to decrease failure rates by means of reduced volumetric wear, which in turn would diminish osteolysis caused by polyethylene wear, and they offered the benefit of enhanced component stability due to the use of larger-diameter femoral heads [4].

In the following years, MoM systems caused high rates of implant failure due to adverse reaction to metal debris (ARMD) ranging from local tissue asymptomatic lesions to serious complications such as osteolysis, solid or cystic pseudo-tumor and necrosis, with high blood levels of Cr–Co and a consequent risk of systemic manifestations [5–7].

As a consequence, the large amount of failure after THA with MoM systems caused a revision rate up to three times higher than other types of prosthesis [8]. Clinical symptoms might be absent, but the growth of pseudotumor can cause manifestations such as pain, swelling, local periarticular destruction and compression neurovascular, gastrointestinal and genitourinary syndromes [9]. For this reason, patients who underwent THA with MoM implants need a careful follow-up and accurate indications to revision [10].

Implants with large-diameter heads, specifically those 36 mm or larger, have been found to enhance stability and reduce the risk of dislocation [11]. However, the positive effects of larger head sizes are offset when exceeding 38 mm, as this results in elevated volumetric metal wear [12, 13]. Furthermore, research has demonstrated a high incidence of hip pain following MoM THA procedures that use a large 38-mm femoral head, in many cases linked to soft tissue impingement of the iliopsoas which may lead to painful tendon irritations [14, 15]. Additionally, patients experiencing pain exhibited substantially elevated levels of serum metal ions compared to those not reporting pain [13, 16].

The aim of this study was to present the long-term outcomes of MoM THA on a large cohort of patients with a follow-up period ranging from 0 to 21 years, to evaluate the impact of variable femoral head sizes on survival rate and to assess whether the MoM implant can have a future role in hip arthroplasty.

## Materials and methods

This study examines all of the MoM THAs that were implanted in Emilia Romagna (ER), Italy, retrospectively. ER is a region in Northern Italy, in which 4459 million inhabitants live. The analysis was done using information from the regional joint register, or Registro dell’Impiantologia Protesica Ortopedica (RIPO). The register was created in 2000 and collected data on all hip, knee, and shoulder replacement surgeries carried out in the Italian Region of ER. There are 69 active orthopaedic units in this area, which can be either public or private. The total amount of population is established to be 4.5 million. Every Unit gave their consent for the data to be gathered. The register is member of the International Society of Arthroplasty Registries and his accuracy of the data collected by this register in 2017 was 97.2%. Italian Social Security numbers were used to identify patients who were residents of the area but had surgery outside of it. Furthermore, any revisions made outside of the area can be tracked. For these reasons we decided to include in the study only patients residing in ER, in order to know exactly the survival of each prosthesis in the cohort.

We decided to include in the study all patient who underwent surgery using MoM THAs between 2000 and 2020. Additionally, in attempting to standardize the sample, we limited the study to patients who had exclusively cementless prostheses. Failures were recorded up to December 31, 2020. The extraction from the database was made on January 22, 2024. All procedures performed on patients living outside ER region were excluded to minimize bias due to loss to follow-up.

To pursue the aim of the study, two groups of patients have been established according to head size. “Small head” refers to sizes under 36 mm, while “Large head” refers to sizes equal to or beyond 36 mm.

Ethics approval was not necessary as the data were collected from a registry in Italy that collects data as standard practice on all patients in their region. Additionally, all data were collected and analyzed in a de-identified format that protects patient privacy.

### Statistical analysis

In both cohort patient and implant features were presented as mean and standard deviation if continuous and as frequency and percentage if categorical. Also causes of revision were presented as rate and percentage.

Indication for surgery, age in class, gender, BMI type of neck were compared using Chi-square analysis, while age was compared using a *t*-test. Differences between the groups were considered statistically significant if the *p* value was less than 0.05. Kaplan–Meier survivorship analysis was performed using revision of at least a component as the endpoint and survival times of unrevised implants as the last observation date (December 31, 2020, or date of death). Further four Kaplan–Meier analysis were conducted with aseptic loosening, prosthesis breakage, metallosis and septic loosening as the endpoints. The Log-Rank Test test was used to compare survivorship between the two groups. The Cox multiple regression model for analyzing survival data was considered. The proportionality hazards assumption was tested by the Schoenfeld residual method.

The statistical analysis was performed using R Core Team (2023). R: A Language and Environment for Statistical Computing. R Foundation for Statistical Computing, Vienna, Austria (https://www.R-project.org/).

## Results

**Fig. 1:**
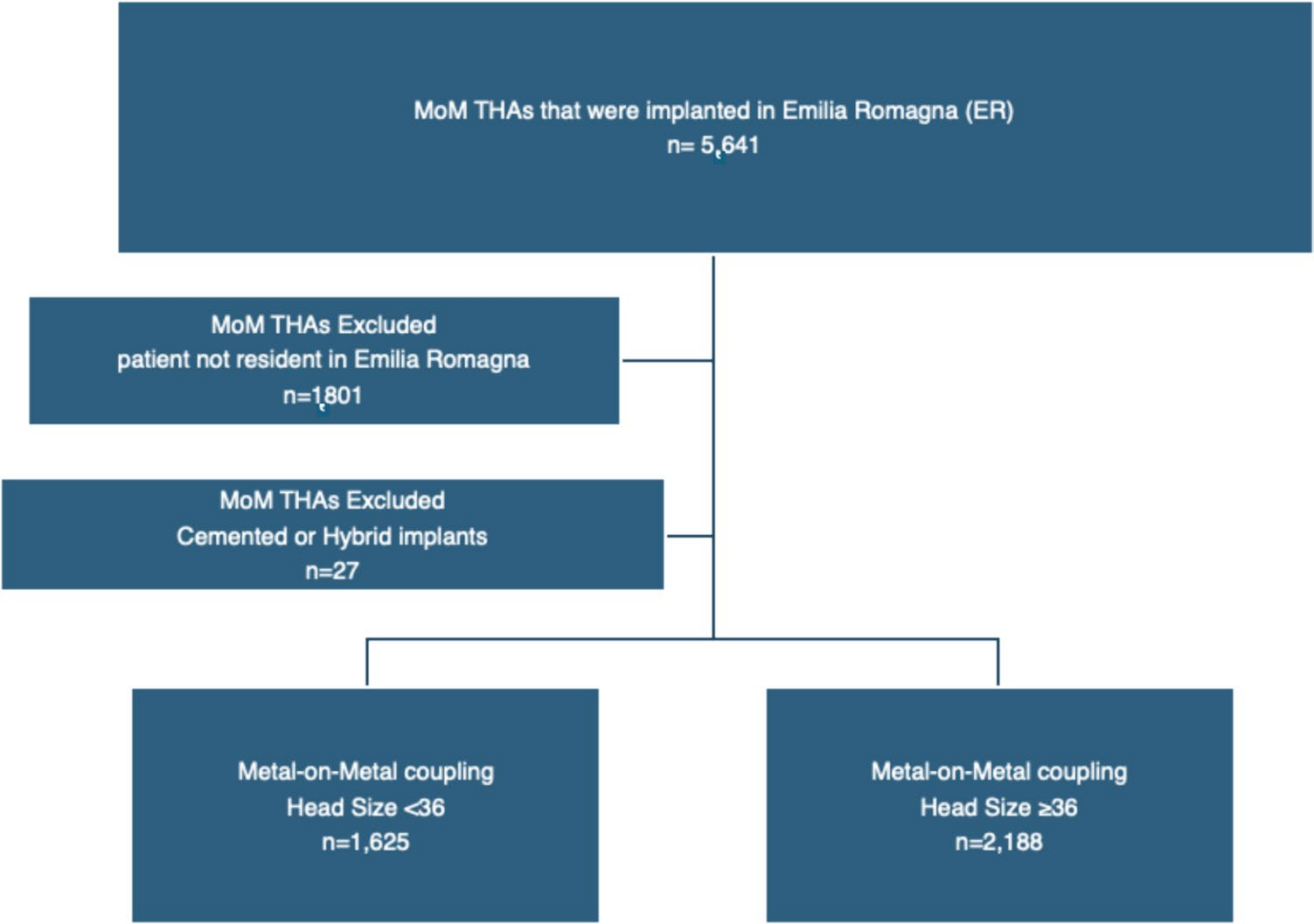
Flow diagram illustrating patients’ inclusion

Throughout the examined time frame, a total of 5641 MoM THAs were registered; according to the exclusion criteria, 3813 THAs were selected and included in the cohort (Fig. 1). The average follow-up period is 12.4 years (interval: 0–21 years). The cohort is composed of 1977 men (51.8%) and 1836 women (48.2%). On the day of surgery, the mean patient age was 60.7 years (range, 15–87 years). None of the prostheses implanted after 2012 that satisfied the inclusion criteria were found.

Table 1 contains all the general information on the patients under examination. The distribution of the prosthesis implanted each year is displayed in Table 2.

**Table 1:** Patients and implant characteristics according to head size

Characteristic	<36, *N* = 1625^a^	≥36, *N* = 2188^a^	*p*-value^b^
Age			<0.001
Median (range)	60 (18, 85)	63 (15, 87)	
Mean (SD)	59 (11)	62 (11)	
Age in class			<0.001
<40	78 (4.8)	74 (3.4)	
40–49	185 (11.4)	215 (9.8)	
50–59	517 (31.8)	551 (25.2)	
60–69	623 (38.3)	736 (33.6)	
70–79	193 (11.9)	520 (23.8)	
80+	29 (1.8)	92 (4.2)	
Gender			<0.001
F	936 (57.6)	900 (41.1)	
M	689 (42.4)	1288 (58.9)	
BMI			0.008
Underweight	12 (1.0)	13 (0.7)	
Normal weight	401 (35.0)	558 (29.7)	
Overweight	523 (45.6)	963 (51.2)	
Obese	210 (18.3)	347 (18.4)	
Unknown	479	307	
Neck			<0.001
Fixed	1613 (99.3)	1739 (79.5)	
Modular	12 (0.7)	449 (20.5)	
Diagnosis			<0.001
Primary arthritis	997 (61.7)	1419 (65.1)	
Sequelae of LCA and DCA	291 (18.0)	317 (14.5)	
Femoral head necrosis (idiopathic, due to dialysis, due to steroids)	107 (6.6)	187 (8.6)	
Femoral neck fracture and sequelae	138 (8.5)	136 (6.2)	
Other	83 (5.1)	120 (5.5)	
Unknown	9	9	

^a^*n* (%)

^b^Welch two sample *t*-test; Pearson’s Chi-squared test

**Table 2:** Total THAs divided by year, according to head size

Year	<36	≥36	Total
2000	168	36	204
2001	191	53	244
2002	221	33	254
2003	238	42	280
2004	244	82	326
2005	183	170	353
2006	181	303	484
2007	85	311	396
2008	53	357	410
2009	52	351	403
2010	6	284	290
2011	2	126	128
2012	1	40	41
Total	1625	2188	3813

**Fig. 2:**
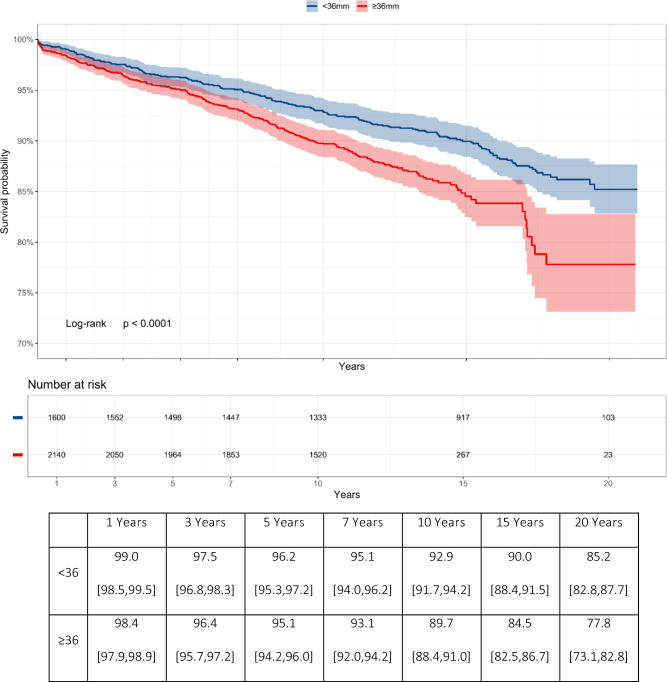
Kaplan–Meier survivorship analysis between groups by Head Size during the various follow-up periods, represented both as percentage of implant survival with ranges and graphically over time

Kaplan–Meier survivorship analysis revealed different survival rates at long-term follow-up of 10, 15, and 20 years among the different diameter heads (Fig. 2). Note the statistically significant difference between the two curves and the differences of patients at risk at 20 years.

Table 3 lists every single explanation why the primary prosthesis failed.

**Table 3:** Revision diagnosis of metal/metal primary total conventional hip replacement by head size

	<36			≥36		
Causes of failure	*N*	IR (%)	% Failure	*N*	IR (%)	% Failure
Cup aseptic loosening	58	3.6	32.6	47	2.1	17.7
Unknown	17	1.0	9.6	43	2.0	16.2
Metallosis	10	0.6	5.6	39	1.8	14.7
Stem aseptic loosening	14	0.9	7.9	32	1.5	12.1
Total aseptic loosening	20	1.2	11.2	16	0.7	6.0
Breakage of prosthesis	15	0.9	8.4	15	0.7	5.7
Septic loosening	15	0.9	8.4	16	0.7	6.0
Dislocation	7	0.4	3.9	21	1.0	7.9
Periprosthetic bone fracture	11	0.7	6.2	17	0.8	6.4
Pain without loosening	4	0.2	2.2	12	0.5	4.5
Other	3	0.2	1.7	4	0.2	1.5
Primary instability	3	0.2	1.7	2	0.1	0.8
Heterotopic bone	1	0.1	0.6	1	0.0	0.4
Total	178	11.0	100	265	12.1	100

The multivariate analysis conducted to determine whether age, gender, or femoral head size could be considered risk factors for conventional primary MoM THA failure is displayed in Table 4. It is shown that only head size can influence the survival rate of this implant. It was found that the only factor affecting MoM implant’s survival rate is the head size.

**Table 4:** Results ﻿of multivariable analysis to detect the influence of risk factor in failure of metal/metal primary total conventional hip replacement

	HR	95% CI	*p*-value
**Age**	0.9	0.5, 1.8	1.06
**Sex**			
F	–	–	
M	0.99	0.8, 1.2	0.96
**Head**			
<36	–	–	
≥36	1.5	1.2, 1.8	<0.001
**Neck**			
Modular	–	–	
Fix	0.9	0.7, 1.2	0.56

*HR* hazard ratio, *CI* confidence interval

**Fig. 3:**
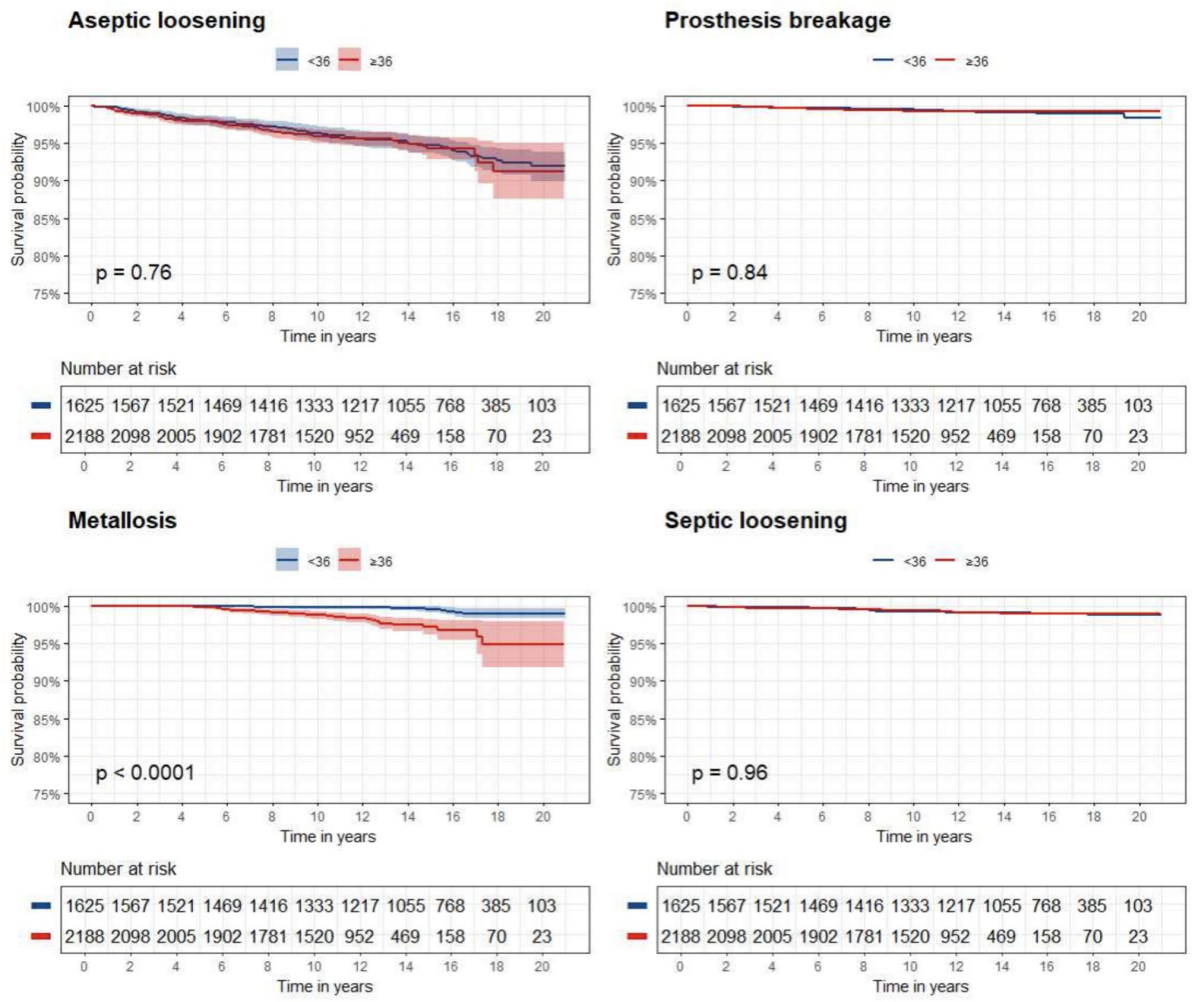
Kaplan–Maier curves of the two groups with different causes of failure as endpoint

Figure 3 shows the Kaplan–Maier curves of small and large head THAs with the four main causes of failure as endpoint.

**Fig. 4:**
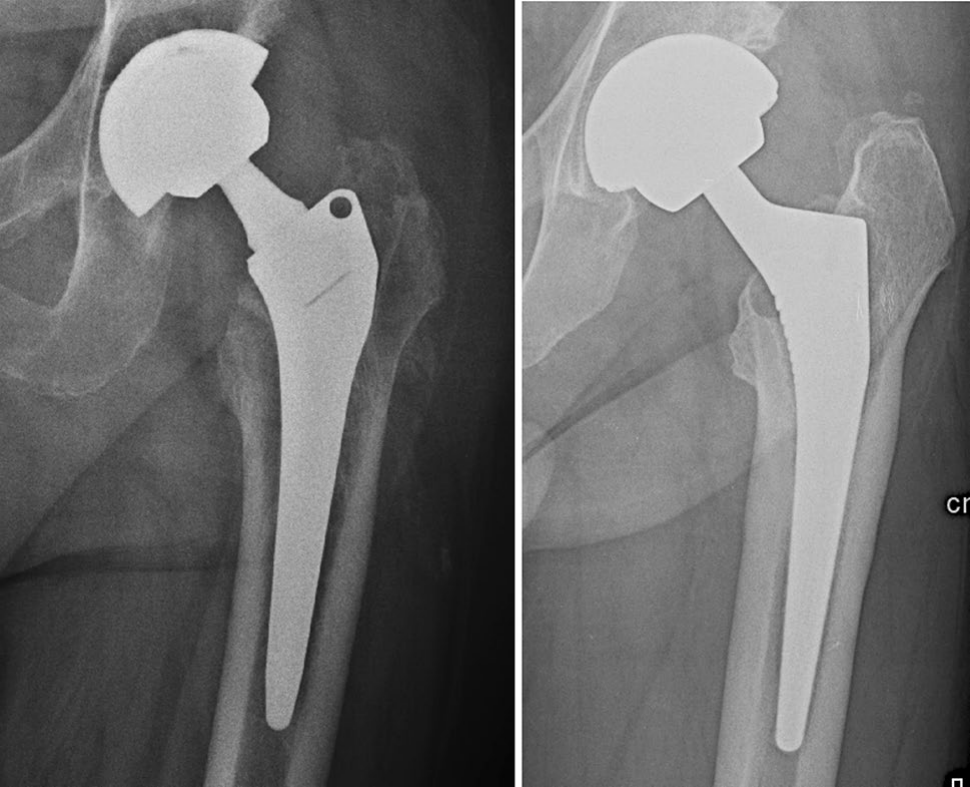
Cup’s orientation of two MoM THAs failed due to metallosis

We identified the MoM prostheses implanted at our Department (Guglielmo da Saliceto Hospital, Piacenza) that failed. A total of 5 THAs with head diameter greater or equal to 36 mm were revised due to metallosis. We retrieved the postoperative X-rays after primary surgeries and used them to compute the cup’s orientation angles. The cup’s orientation was >50° in 3 cases, while it was <30° in 2 THAs (Fig. 4).

## Discussion

MoM coupling has almost disappeared in THA; nowadays, it is limited to use in resurfacing hip prostheses [17]. Historically, MoM prostheses were used in young patients with high functional demands, above all due to the lower wear of the mechanical surfaces, as well as the lower risk of component breakage. The use of large heads was instead associated with an increase in elasticity, reduced pressure and impingement, increase in proprioception [18]. These qualities appeared to have the potential to improve clinical outcomes, broaden the range of motion, ensure implant’s stability, and reduce the dislocation rate without jeopardizing implants longevity [19]. However, these advantages over prostheses with different couplings have been debated [20].

Over the last 15 years, numerous scientific studies have reported disastrous clinical outcomes, also supported by information gathered from the main global prosthetic registries [21–23]. This has led to a growing abandonment of this type of prosthesis, giving way to more reliable couplings [20–22]. However, distinctions between MoM prostheses are required because different scenarios can arise depending on head size. In fact, with larger head sizes, there are a number of issues to be aware of, such as the generation of wear debris at the trunnion-bore interface, wear-related adverse soft tissue reactions, increases in frictional torque, soft tissue impingement, and elevated metal ions levels [14, 15, 24].

Within the examined cohort of patients, the size of the head was found to be a determining factor in the survival of the implant, as it well shown by other national registries [11, 25]. It’s remarkable to observe that starting from eight years after primary surgery the two curves differ significantly.

A very important fact to consider is that in the early 2000s the trend was to use small-head MoM prostheses, while from 2006 onwards the trend reversed itself, with small-head MoMs decreasing drastically (Table 2). Consequently, it appears that many large-head MoMs failed in the first few years after implantation, confirming what reported by Van Lingen et al. in 2022 [26]. About head size, our results partially confirm what has been reported in other prosthetic registries, such as the Australian Orthopaedic Association National Joint Replacement Registry (AOANJRR) and the English National Joint Registry (NJR). The AOANJRR states that from 1999 to 2015, 5807 THAs with a femoral head of 32 mm or less and 16,300 THAs with a femoral head greater than 32 mm were implanted; of these, 511 small head THAs (8.8%) and 3959 (24.3%) of large head THAs failed and required revision surgery [11]. On the other hand, according to the NJR, the lowest revision rates for uncemented MoM THAs was seen in heads with a size of 28 mm; while the worst was registered by 38–48 mm heads; furthermore, there was a statistically significant difference between large and small heads’ revision rate in this registry [25].

Analyzing the causes of failure of prostheses with large heads, our data do not confirm that their use is a protective factor against dislocation, contrary to what has previously been demonstrated by other studies [27]. The percentage of dislocation on large head THAs is significantly higher than that on small head ones in the same sample, even though it is similar to those reported in the literature [28]. We can therefore hypothesize that the cup could also play an important role. Currently, the use of dual mobility cups appears to be a safer method of preventing implant dislocation, especially in individuals who are most at risk such as older age, high BMI, neurological disorders, previous spinal surgery, psychiatric disease [29–31].

Moreover, regarding the inclination of the cup, position it within the so-called Lewinnek safe zone is thought to provide protection against wear and metallosis, as well as from early loosening, dislocation, suboptimal outcomes [32]. Especially in MoM THAs, edge loading is held responsible for increased wear and may result in unforeseen clinical issues and lower tribological performance, metallosis, unfavorable peri-prosthetic tissue reactions like pseudotumours, and increased local and overall articulation wear [33]. In addition, Miguela Alvarez et al. detected that acetabular cup’s verticality has a moderate relationship with the rise in blood ions, while having a weak and inverse correlation with head size [34]. Despite the limitations of our data, we can verify through analysis that cup’s orientation outside of Lewinnek safe zone could be a major risk factor for the development of metallosis. However, additional research will be required to validate these preliminary results.

Regarding metallosis, there is a statistically significant difference in the survival of the two investigated groups, although the diameter of the head does not appear to affect the main causes of implant failure (Fig. 4). This finding supports what has been reported by previous studies regarding the greater tendency of large heads to create friction, releasing metal ions with an inflammatory reaction in soft tissues (ALTR, ARMD, pseudotumor) [16, 20, 26]. On the other hand, <36 mm head THAs have a major risk of aseptic mobilization of the cup, confirming what previously reported by Moon et al.; in their study on 28 mm head MoM THAs with a mean follow up of 20 years, 10/92 implants were revised for aseptic loosening of the cup [35].

The Cox regression analysis showed that head size is the only variable influencing the risk for revision for any cause, the risk for revision increase for head size ≥36. Nevertheless, gender, age and neck modularity have not to be consider risk factors for large head implant failure.

Regarding modular necks, the Cox multivariate analysis did not show a statistically significant higher risk either on the total cohort (*p* value 0.13). Indeed, in our Department modular necks were widely used (50.3% of the total THAs), but metallosis occurred more frequently in prostheses with a fixed neck (+50%). This data confirms the hypothesis that the presence of additional modularity within the prosthesis is not the cause of a greater susceptibility to the development of metallosis, ALTR and ARMD, as already highlighted in previous studies [36].

Many MoM implant failures are linked to the so-called “trunnionosis” of the head-neck taper connections [37, 38]. This condition is defined as the current presence of wear and corrosion and resulting from a long-term action of wear and corrosion at the stem modular junction, which over time weakens the implant and changes the surrounding soft tissue biologically [38–40]. Component breakage, disassembly of the femoral components, and substantial trunnion material loss are among the most dramatic occurrences of trunnionosis, also known as gross trunnion failures (GTF) [41]. Numerous circumstances can lead to GTF, and the most significant risk factors identified in the literature are male gender, body mass index greater than 30, high offset femoral component, femoral head >36 mm, long neck length, implant malpositioning and intraoperative component damage [42]. Focusing on head size, previous studies discovered that stresses at the trunnion-head junction significantly rise in case of a large head is employed [43]. Increased stresses and micro-motion may cause metal ion release, tribocorrosion, weakening of the trunnion, fracture and early collapse of the implant, even without traumatic events [43, 44].

### Strength and limitations of the study

This study stands out from many other researches since it provides a very long follow-up (up to 21 years). Furthermore, the examined data is accurate and unbiased as it comes from a certified prosthetic register; that is, we are able to report on the survival of each and every single prosthesis included in the sample; precisely for this reason, all patients lost to follow up were excluded from the study. On the other hand, the study has some limitations, starting from its retrospective design which is designed to analyze pre-existing data and does not allow us to decide what information to collect; in this regard, an important data that is missing from the study is the blood’s metal ion concentration; we hope to be able to delve deeper into this aspect in a future study.

## Conclusions

In conclusion we can state that MoM implants do not have the same reliability as other couplings, considering the significantly greater failure/complication rates. However, nowadays there are still many patients with a MoM THA, and knowledge of the risks, also and above all those linked to the type of components used (such as the size of the head), becomes fundamental for establishing the right type of follow-up to the patient and, in the worst cases, recognize any complications early.

Large head MoM THAs are burdened by higher failure rates and are more inclined to develop adverse reactions in the soft tissues (ALTR, ARMD, pseudotumor); on the other hand, small head ones are more prone to suffer from aseptic loosening of the cup. Contrary to what other studies have stated, we did not find a reduction in dislocations in the large head subgroup.

Finally, there were no associations found between the failure rate and age, gender, or modular neck usage.

## Data Availability

The data that support the findings of this study are available from the corresponding author upon reasonable request. Some data are publicly available at RIPO (http://ripo.cineca.it/authzssl/index.htm).
